# Oral food challenge to wheat: a near-fatal anaphylaxis and review of 93 food challenges in children

**DOI:** 10.1186/1939-4551-6-14

**Published:** 2013-08-21

**Authors:** Antonella Cianferoni, Karishma Khullar, Rushani Saltzman, Joel Fiedler, Jackie P Garrett, David R Naimi, Jonathan M Spergel

**Affiliations:** 1 The Department of Pediatrics, Division of Allergy and Immunology, The Children's Hospital of Philadelphia, 3550 Market Street, Suite 3054, Philadelphia, PA 19104-4399, USA; 2 Current address: Pediatrics Northwest Asthma & Allergy Center University of Washington School of Medicine, Seattle, WA, USA

## Abstract

**Background:**

Wheat allergy is among the most common food allergy in children, but few publications are available assessing the risk of anaphylaxis due to wheat.

**Methods:**

In this study, we report the case of near-fatal anaphylaxis to wheat in a patient undergoing an oral food challenge (OFC) after the ingestion of a low dose (256 mg) of wheat. Moreover, for the first time, we analyzed the risk of anaphylaxis during an OFC to wheat in 93 children, compared to other more commonly challenged foods such as milk, egg, peanuts, and soy in more than 1000 patients.

**Results:**

This study, which includes a large number of OFCs to wheat, shows that wheat is an independent risk factor that is associated with anaphylaxis requiring epinephrine administration (Odds Ratio [OR] = 2.4) and anaphylaxis requiring epinephrine administration to low dose antigen (OR = 8.02). Other risk factors for anaphylaxis, anaphylaxis requiring epinephrine administration, and anaphylaxis to low dose antigen was a history of a prior reaction not involving only the skin (OR = 1.8, 1.9 and 1.8 respectively). None of the clinical variables available prior to performing the OFC could predict which children among those undergoing OFCs to wheat would develop anaphylaxis or anaphylaxis for low dose antigen.

**Conclusion:**

This study shows that wheat is an independent risk factor that is associated with anaphylaxis requiring epinephrine administration and anaphylaxis requiring epinephrine administration to low dose antigen.

## Introduction

The recommended evaluation for food allergy (FA) includes a detailed history and physical examination, followed by selected *in vivo* and *in vitro* tests based on the history (1). Oral food challenges (OFCs) are performed either because the food allergy is not supported by history, because there is a discrepancy between history and test results or because newly developed tolerance need to be established [1]. The demand for OFCs for FA evaluation has greatly increased in the last decade due to an increased prevalence of FA [2] and an increased number of elimination diets started solely on the basis of commercially available *in vitro* testing [3]. OFCs are commonly associated with inherent risks, as up to 28% of OFCs have systemic reactions [4]. However, for the most part, they are perceived to be safe and hence, they are often done in an outpatient office setting not necessarily located in close proximity to a hospital [5]. The increased need for OFCs has created a need to identify which patients are at the greatest risk to develop anaphylaxis during an OFC, so that the correct location of OFC performance can be determined and appropriate counseling to the family on risk associated with OFC can be reviewed [5-7].

Wheat has been increasingly reported to be a risk factor for exercise-induced anaphylaxis [8]. Water insoluble omega-5-gliadin (Tri a 19) has been identified as a major allergen in Finnish subjects with Food-Dependant Exercise-Induced Anaphylaxis (FDEIA) [9,10]. However, in studies on food challenges, wheat has never been reported as a specific risk factor for developing anaphylaxis [3-5,7]. Most studies have found that wheat challenges present a rate of failure similar to other foods (30-50%), with use of epinephrine only in fraction of cases (10-20%) [4,5].

In this study, we report a case of near-fatal anaphylaxis to wheat in a patient undergoing an OFC after the ingestion of a low dose of wheat. Moreover, for the first time, we will analyze the risk of anaphylaxis during a food challenge to wheat; compared to other more commonly challenged foods such as milk, egg, peanuts, and soy. This study contains one of the largest reported numbers of food challenges to wheat and specifically analyzes wheat as a risk factor for anaphylaxis during OFCs.

## Methods

### Study population and oral challenges

We performed a retrospective analysis of children who underwent OFCs to wheat, soy, milk, egg, and peanut at The Children’s Hospital of Philadelphia (CHOP) Pediatric Day Medicine unit from August 2004 to December 2010. Oral food challenges (OFCs) were performed either because a food allergy was not supported by history (i.e. the food has never been eaten before or was previously tolerated and stopped because of positive skin testing or detection of specific serum IgE for the food) or because newly developed tolerance needed to be established. None of the reported OFCs were done prior to and with the intent to initiate an oral desensitization protocol. Clinical data of patients’ serum wheat, soy, milk, egg white, and peanut–specific IgE antibody levels (sIgE), skin prick test (SPT) results, previous history of systemic reaction, history of asthma or eczema, and oral challenge outcomes of children were collected. All patients had either skin prick testing or specific serum IgE (ImmunoCap) performed in the 6 months prior to the OFC. Parents of the child described in the case report provided written consent for data to be used in this publication. All investigations were approved by the CHOP Internal Review Board. (See also supplemental material). OFCs were performed by starting with a dose of 0.1 mL, followed by 0.5, 1, 2.5, 5, 10, 30, and 60 mL, 120 ml, and 240 ml for liquid foods (milk). For solid foods (peanut, egg powder, milk powder, wheat and soy), the challenge doses administered were 125 mg, 250 mg, 500 mg, 1gm, 2 gm, 4 gm, 8 gm, and ad lib (minimum of 8 gm). In select cases a lower starting dose (20–60 mg) was chosen for very high risk kids. Each dose was administered with an interval of 15 to 20 minutes until ad lib doses were reached or the patient experienced a reaction within 2 hours of the last dose. Challenges were stopped for gastrointestinal reactions, respiratory cardiovascular or neurologic symptoms, non-contact cutaneous reactions or multi-system reactions. All providers followed the same standardized protocol as described. All children held antihistamine use for at least 5 days prior to OFC.

### Classification of reactions

Reactions on presentation and challenges were classified as cutaneous, respiratory, gastrointestinal, cardiovascular, neurologic, multiple-organ system [11,12]. Cutaneous reactions included urticaria, erythematous flushing, cutaneous angioedema, or flaring of atopic dermatitis on non-contact areas. Respiratory reactions encompassed rhinitis, sneezing, voice change, throat tightness, dyspnea, cough, wheeze, shortness of breath, or tachypnea. Gastrointestinal reactions consisted of abdominal pain, emesis, or diarrhea. Cardiovascular reaction included hypotension and neurologic reactions included fainting [11,12]. Medications were administered immediately on detection of an allergic reaction, and the decision to administer was based on clinical judgment. The reactions for which epinephrine was given were based on current clinical definition of anaphylaxis [13]. Although multisystemic reactions in an outpatient setting should be treated with epinephrine [11,14], in a hospital setting under the supervision of an expert physician treating allergic reactions, epinephrine is used with more stringent criteria of anaphylaxis [13,15,16]. Multisystemic reactions characterized by mild skin reactions and transient abdominal pain or 1 episode of mild vomiting, or mild transient rhinitis, were classified as multi-systemic but were not treated in all cases with epinephrine. Patients were observed for at least 4 hours after an allergic reaction.

Patients were divided into 5 categories: positive OFC (any reaction), OFC that resulted in multiorgan involvement (Multisystemic), OFC that resulted in multiorgan involvement after low dose ingestion (Multisystemic <1 g), OFC that resulted in epinephrine intramuscular (i.m.) administration (Epinephrine), and OFC that resulted in epinephrine i.m. administration after low dose ingestion (Epinephrine <1 g) [11,12].

### Skin testing and serum IgE

SPTs were performed by the prick method using commercial extracts (Greer Laboratories, Lenoir, NC) and bifurcated needles. Maximal wheal and flare diameter was measured at 15 minutes (12). Serum samples were analyzed for egg white, milk and peanut–sIgE antibody concentrations by using the Pharmacia CAP system FEIA (Pharmacia and Upjohn Diagnostics, Uppsala, Sweden). The lower limit of detection was 0.35 kU/L. (see Supplemental material for details).

### Statistical methods

The relationships between the different types of OFC outcomes and other characteristics in both groups of children were analyzed by calculating the odds ratio and confidence intervals using a univariate or multivariate logistic regression [17].

All tests were performed with STATA (version 11.0 for Windows; STATA Inc, College Station, TX,USA). (Additional file 1 information is in supplemental material).

## Results

### OFC analysis

History of asthma, eczema, type of previous reaction, history of prior ingestion, sIgE and SPT-wheal measures to specific foods, and the outcome of food challenges in 1187 open OFCs to wheat, milk, egg, soy or peanuts were analyzed. The demographics of the study population are reported in Additional file 1 Table S1E. More than half of the children had co-morbidity with eczema (53%) and/or asthma (56%). The group consisted primarily of males (70%) around 5 years of age (mean age 4.8 years: range 6 months to 20 years). Skin tests were done in 1172 patients, and specific IgE for specific allergens were available in 755 patients. Given both the high cost of co-pay in most insurance plans for specific serum IgE and needle phobia, a significant proportion of patients opted to do food challenges without obtaining sIgE first. In 15 patients, only serum IgE were obtained, as skin testing was not possible for multiple reasons, including inability to stop antihistamines, parental refusal, and severe eczema. There were 3 patients in the milk group who did not have skin testing (Serum IgE Ku/L respectively 1; 1.18; 9), 5 in the egg group (Serum IgE Ku/L respectively 3.22; 2; 2.42; 4.72; 2.22), 4 in the peanut group (Serum IgE Ku/L respectively 10; 0.66, 1, 2), 1 in the wheat group (Serum IgE Ku/L 44), and 2 in the soy group (Serum IgE Ku/L respectively 1.13 and 4). This is in line with current guidelines, which state that sIgE tests and skin testing are useful to identify foods which may potentially provoke IgE-mediated food-induced allergic reactions. However the same guidelines state that positive skin testing and sIgE when used alone are not diagnostic of FA. Furthermore, the guidelines do not recommend the use of sIgE or SPT as the sole guide to determine whether or not to perform an OFC [11]. Hence, OFCs are often decided based on clinical history and necessity, as well as the benefit/risk ratio to reintroduce a food. Particularly for those foods such as milk, egg, and wheat, which are a major staple of the Western diet, a higher risk of failing OFCs is generally accepted by parents and physicians [15]. The OFCs we have analyzed, as well as the one reported in the clinical case, were performed for clinical and not research purposes. Hence, the work-up was largely based on current guidelines.

The mean wheal and flare in mm for all patients was 6.1 mm (range 0-25 mm) and 16.3 mm (range 0-45 mm) respectively. The mean sIgE for specific food allergens were 7.1 ± 16.3 kU/L (range 0–100 kUI/L). Reported outcomes of wheat OFCs based on sIgE and skin prick test levels are given in Additional file 1 Table S2E.

Patients who underwent the challenge for wheat tended to be younger, had smaller skin tests but higher levels of sIgE, had asthma less often, and had a history of prior ingestion or prior reaction to the food tested compared to those children who presented for food challenges to all the other foods (Table 1). Those children who failed the challenge to wheat were younger and had smaller skin tests, but higher levels of sIgE. Younger age and smaller skin tests were also noted in those who had multisystemic reaction and in those who needed intramuscular epinephrine. (Table 1 and Additional file 1 Table S3E). This is not surprising, as younger children tend to have smaller skin prick test. The higher sIgE can be explained by the fact that sIgE cut-offs for wheat have poor specificity and sensitivity in predicting OFC outcomes and is generally not considered a good screen to decide who needs to undergo OFC to wheat [4,18].

**Table 1:** Characteristics of children undergoing open oral food challenge (OFC) to wheat vs those undergoing OFC to other foods

	**All OFC**			**OFC positive**			**Multisystemic reactions**			**Epinephrine**		
	**Wheat (93)**	**Other (1094)**	**p**	**Wheat (39)**	**Other (489)**	**p**	**Wheat (28)**	**Other (330)**	**p**	**Wheat (17)**	**Other (135)**	**p**
Age (mean ± SE)	3.6 ± 2.4	4.9 ± 0.1	**<1X10**^**-4**^	3.4 ± 0.3	5.1 ± 0.1	**<1X10**^-**4**^	3.5 ± 0.33	5.2 ± 0.33	**<1X10**^-**3**^	3.6 ± 0.5	5.6 ± 2.4	**<1X10**^-**3**^
Sex (female) (5)	28 (30%)	330 (30%)	**ns**	11 (29%)	148 (30%)	**ns**	6 (21%)	88 (26%)	**ns**	2 (12%)	32 (24%)	**ns**
Wheal mm (mean ± SE)	5.1 ± 2.6	5.9 ± 0.1	**<0.05**	5.5 ± 0.5	7.6 ± 0.2	**<1X10**^-**3**^	5.5 ± 0.54	7.8 ± 0.21	**<1X10**^-**3**^	5.7 ± 0.8	8.1 ± 0.4	**<0.05**
Flare mm (mean ± SE)	12.1 ± 15.8	16.1 ± 0.3	**<1X10**^-**3**^	12.5 ± 0.9	19.4 ± 0.4	**<1X10**^-**4**^	12.5 ± 1.01	20.1 ± 0.46	**<1X10**^-**4**^	12.6 ± 1.4	21.1 ± 0.8	**<1X10**^-**3**^
sIgE (KUI/L) (mean ± SE)	19.9 ± 22.8	6.5 ± 0.6	**<1X10**^-**4**^	29.7 ± 10.5	10.1 ± 1.1	**<1X10**^-**3**^	17.6 ± 9.1	10.5 ± 1.3	**ns**	46.1 ± 5.1	12.7 ± 2.6	**ns**
Prior Ingestion	57 (61%)	800 (73%)	**<0.05**	28 (72%)	361 (74%)	**ns**	19 (68%)	241 (73%)	**ns**	11 (65%)	91 (67%)	**ns**
Prior Reaction	50 (54%)	712 (65%)	**<0.05**	25 (64%)	340 (69%)	**ns**	16 (57%)	223 (67%)	**ns**	11 (65%)	81 (60%)	**ns**
Prior Reaction not skin	42 (45%)	527 (48%)	**ns**	22 (56%)	269 (55%)	**ns**	14 (50%)	197 (60%)	**ns**	12 (71%)	81 (60%)	**ns**
Asthma History	38 (41%)	581 (53%)	**<0.05**	21 (54%)	292 (60%)	**ns**	17 (61%)	204 (62%)	**ns**	10 (58%)	75 (55%)	**ns**
Eczema History	48 (52%)	523 (48%)	**ns**	23 (59%)	238 (49%)	**ns**	17 (61%)	157 (47%)	**<0.05**	10 (58%)	70 (51%)	**ns**
Time of Prior Exposure ≥1 year	34 (36%)	596 (54%)	**<1`X10**^-**2**^	20 (52%)	311 (63%)	**ns**	16 (57%)	202 (61%)	**ns**	9 (53%)	86 (63%)	**ns**

We performed a univariate logistic regression to see if any clinically available characteristics could help to identify children who were at higher risk to develop multisystemic reactions or multisystemic reactions for low dose of allergen with or without the use of epinephrine during a wheat challenge. Having a history of asthma and a previous reaction were associated with multisystemic reactions (Table 2).

**Table 2:** Univariate logistic regression to predict outcome of OFC to wheat

	**OFC positive**			**Multisystemic**			**Multisystemic <1 g**			**Epinephrine**			**Epinephrine <1 g**		
	**OR**	**SE**	**p**	**OR**	**SE**	**p**	**OR**	**SE**	**p**	**OR**	**SE**	**p**	**OR**	**SE**	**p**
Age	0.93	0.1	**0.5**	0.97	0.1	**0.8**	1.6	0.84	**0.3**	1.1	0.1	**0.9**	1.7	0.2	**0.7**
Sex (female) (5)	0.85	0.4	**0.7**	0.2	0.2	**0.08**	0.2	0.2	**0.2**	0.2	0.2	**0.08**	0.16	0.2	**0.1**
Wheal mm	1.1	0.2	**0.2**	1.1	0.1	**0.15**	0.9	0.2	**0.5**	1.1	0.1	**0.3**	1.1	0.7	**0**.**9**
Flare mm	1.1	0.1	**0**.**6**	1.01	0.03	**0.3**	0.9	0.1	**0.8**	1.01	0.1	**0.7**	1.1	0.1	**0**.**6**
sIgE (KUI/L) (mean ± SE)	1.02	0.1	**0**.**2**	0.99	0.02	**0.6**	n/a	n/a	**n**/**a**	1.02	0.1	**0.2**	n/a	n/a	**n**/**a**
Prior Ingestion	2.2	0.9	**0.08**	1.2	1.5	**0.4**	0.4	0.45	**0.4**	1.2	0.6	**0.8**	0.45	0.45	**0.4**
Prior Reaction	2.1	0.9	**0.09**	3	1.5	**0.028**	1.1	1.16	**0.9**	2.4	1.4	**0.1**	1.28	1.12	**0.8**
Prior Reaction not skin	2.2	0.94	**0.06**	1.4	0.6	**0.5**	0.6	0.6	**0.6**	2.2	0.94	**0.06**	0.6	0.6	**0.6**
Asthma History	2.53	1.1	**0.03**	3.2	1.9	**0.048**	1.6	2.3	**0.7**	2.4	1.3	**0.1**	1.28	1.1	**0.8**
Eczema History	1.6	0.7	**0.2**	1.5	0.9	**0.5**	1.28	1.4	**0.9**	1.4	0.8	**0.5**	1.28	1.1	**0.8**
Time of Prior Exposure ≥1 year	3.1	1.3	**0.01**	3.1	2.7	**0.2**	2.33	2.1	**0.3**	2.3	1.3	**0.1**	2.33	2.1	**0.3**

We then analyzed clinically available characteristics that were associated with the 5 studied categories (Table 3). Forty-five percent of the challenges were positive, with the following failure rates for each food: wheat 39 of 93 (42%), soy 26 of 114 (23%), milk 144 of 290 (50%), egg 190 of 409 (47%), and peanut 130 of 283 (46%). Soy challenges were significantly associated with lower risk of positive OFC and milk with higher risk. As previously reported [7], other risk factors for a positive OFC were older age, larger wheal and flare of SPT, higher sIgE levels, a history of prior reaction and prior reaction not limited to the skin, a personal history of asthma, and a prior ingestion of the food more than one year ago. Risk factors for OFCs resulting in multisystemic reactions were a milk challenge, older age, larger wheal and flare of SPT, higher sIgE levels, a history of prior reaction and prior reaction not limited to the skin, a personal history of asthma, and prior ingestion of the food more than one year ago. A risk factor for OFCs resulting in multisystemic reactions for low dose antigen was undergoing a peanut challenge. Risk factors for reaction requiring epinephrine i.m were a peanut or wheat challenge, larger SPT sizes, higher specific IgE levels, male sex, older, and prior reaction not limited to the skin. Risk factors for anaphylaxis that required epinephrine i.m. use at low dose of antigen were peanut or wheat challenge and a prior reaction not limited to the skin, whereas egg food challenges were associated with the least risk of developing anaphylaxis for low dose antigen (Table 3).

**Table 3:** Univariate logistic regression to predict outcome of OFC to any food

	**OFC positive**		**Multisystemic**		**Multisystemic <1 g**		**Epinephrine**		**Epinephrine <1 g**	
	**OR**	**p**	**OR**	**p**	**OR**	**p**	**OR**	**p**	**OR**	**p**
Wheat	0.9	**0.6**	1.2	**0.6**	1.4	**0.5**	2.1	**0.003**	4.1	**0.004**
Peanut	1.11	**0.6**	1.2	**0.4**	2.1	**0.03**	1.16	**0.01**	1.9	**0.03**
Milk	1.3	**0.04**	1.3	**0.04**	0.65	**0.2**	0.94	**0.9**	0.48	**0.1**
Egg	1.1	**0**.**4**	0.92	**0.5**	0.73	**0.3**	0.7	**0.08**	0.48	**0.02**
Soy	0.33	**<0.0001**	0.32	**<0.0001**	0.28	**0.1**	0.48	**0.06**	0.34	**0.35**
Age	1.1	**0.02**	1.05	**0.012**	1.02	**0.6**	1.1	**0.004**	1.01	**0.3**
Sex (female) (%)	0.99	**0.9**	0.76	**0.056**	0.85	**0.6**	0.62	**0.02**	0.80	**0.5**
Wheal mm	1.3	**<0.0001**	1.2	**<0.0001**	1.04	**0.5**	1.2	**<0.0001**	1.1	**0.2**
Flare mm	1.1	**<0.0001**	1.07	**<0.0001**	1.06	**0.6**	1.1	**<0.0001**	1.1	**0.1**
sIgE (KUI/L) (mean ± SE)	1.02	**<0.0001**	1.01	**<0.0001**	0.99	**0.6**	1.02	**<0.0001**	1.01	**0.2**
Prior Ingestion	1.17	**0.2**	1.07	**0.6**	0.7	**0.2**	0.74	**0.1**	0.7	**0.2**
Prior Reaction	1.99	**<0.0001**	1.5	**0.007**	0.7	**0.2**	0.85	**0.3**	0.7	**0.2**
Prior Reaction not skin	1.7	**<0.0001**	1.9	**<0.0001**	1.6	**0.1**	3.26	**<0.001**	3.75	**<0.02**
Asthma History	1.6	**<0.0001**	1.7	**<0.0001**	1.28	**0.8**	1.2	**0.3**	1.28	**0.8**
Eczema History	1.05	**0.6**	1.03	**0.7**	1.28	**0.8**	1.2	**0.3**	1.28	**0.8**
Time of Prior Exposure ≥1 year	2.8	**<0.0001**	2.5	**<0.0001**	0.9	**0.7**	1.3	**0.1**	0.9	**0.7**

A multivariate logistic regression analyzing the independent weight of the six factors associated with both multisystemic reactions and multisystemic reactions for low dose antigen with or without the use of epinephrine i.m. (wheat challenge, peanut challenge, soy challenge, age, wheal size, and prior reaction not limited to the skin) showed that peanut challenges put patients at risk of anaphylaxis for low dose antigen (regardless of the use of epinephrine) and wheat challenges put patients more at risk of reactions requiring epinephrine, especially after low dose antigen (OR = 8.02).

Having a prior reaction not limited to the skin and larger skin testing were independently associated with multisystemic reactions and multisystemic reactions for low dose antigen and use of Epinephrine.

## Discussion

The demand for OFC for FA evaluation has greatly increased in the last decade due to an increased prevalence of FA [2,19] and the increased number of elimination diets started solely on the basis of the commercially available *in vitro* testing [3]. However, up to 28% of OFCs are associated with systemic and potentially life-threatening reactions [4]. Stratification of the risk of OFC is therefore becoming increasingly important to choose the right location of OFC performance and also to better counsel the family on risk associated with OFC. Previous studies examining the relationship of skin prick tests (SPT) [20-22] or food-specific serum IgE levels (sIgE) [4,21,23,24] and challenge outcome show that both SPT and sIgE tests *individually* are very useful in predicting those patients who have a risk to fail the OFC [25]. Indeed many studies have determined “cut-off” levels for specific serum IgE and skin prick tests size that predict 95% or 50% of the food challenges reactions for some food allergens such as milk, egg, peanut, tree nuts, sesame seed and fish . However far less clear are those risk factors that predispose to develop a severe life threatening reaction after OFC. So far, sIgE and SPT have not been shown to be able to predict severe reactions after OFC when used in isolation [4,26]. Indeed, other factors such as history of asthma, older age or previous history of multiorgan-system reaction have been reported to be associated with more severe reactions in children with FA but not as factors predicting the severity of the reaction induced by OFC [12,27-30]. However, there is a wide agreement that although higher levels of specific IgE increase the likelihood of failing an OFC, it does not predict the severity of the reaction [4,6,30,31]. Two recent papers have suggested that a more complex model incorporating test results and clinical history had a better the predictive ability compared with sIgE and SPT used either alone and in combination [6,7]. Other studies have also shown a challenge to peanut to be a risk factor for more severe reactions requiring epinephrine use [5].

Food allergy to wheat (both IgE and non-IgE mediated) is well-known and manifests with a variety of symptoms that include atopic dermatitis exacerbations [24,32-34], exercise-induced anaphylaxis [8], eosinophilic esophagitis [35], baker’s asthma [36], and celiac disease [37]. The prevalence of clinically relevant wheat allergy is not well established, but is estimated to be less than 0.5% to 1%, with most children outgrowing it by age 16 [18], despite the fact that positive SPTs have been reported in the general pediatric population in more than 3% of children [38]. It also appears that IgE against different epitopes may be responsible for different phenotypes of wheat-related allergy as well as its severity [36,39-46]. Indeed, recently there have been two reports that indicate that omega-5 gliadin predicts for severe reactions in OFC to wheat [47,48]. Furthermore, wheat has been also increasingly reported to be a risk factor for exercise-induced anaphylaxis [8]. Water insoluble omega-5-gliadin (Tri a 19) has been identified as a major allergen in Finnish subjects with Food-Dependent Exercise-Induced Anaphylaxis (FDEIA) [9,10].

Most case series on OFCs report no concern for specific severity of reaction to wheat, and these challenges are generally considered less dangerous than milk, egg, and peanut [3-5,49]. In no cases of fatal or near-fatal anaphylaxis has wheat been reported as a cause [27,50,51]. However werecently treated a 7 year old female who had near fatal anaphylaxis to wheat after OFC. The patient was new to our practice as she moved from out of state and came for an evaluation of multiple food allergies and failure to thrive. She had a history of eczema as an infant and multiple food allergen triggers had been identified. The first ingestion of cow’s milk and egg had caused eczema flares, urticaria, and lip and eyelid angioedema. As part of the eczema and food allergy evaluation in infancy she was found to be positive per parental report to milk, egg, wheat, barley, shellfish, fish, treenuts, and peanuts and was currently avoiding all those foods. When she came to our practice she had a very restricted diet consisting of meats, fruits, vegetables, berries, rice pasta, and soy butter. Her growth was poor. At 7 years of age she had a height of 112 cm (3^rd^ percentile) and a weight of 16.20 kg (0.22nd percentile). We did not have any record of the data (SPT and/or specific IgE) obtained with her initial diagnosis of wheat. The patient had an Immunocap of 65 KU/L for wheat obtained 16 months prior to the OFC.

From the time of diagnosis in infancy, she only had limited accidental exposures to wheat, never as an isolated ingredient. Specifically, at age 1.5 years, she ingested macaroni and cheese and developed hives on her lower extremities which responded to oral diphenhydramine. At age 2.5 years, she had a contact reaction to a brownie, where she developed facial hives which resolved with diphenhydramine. Both foods contained also milk and egg to which she had previous reactions. She had one episode of anaphylaxis at age 3.5 years after the ingestion of seasoned fries which contained both wheat and egg allergens. She developed cough and was given epinephrine for this reaction. Skin prick testing obtained about 3.5 months prior to the OFC included SPT (Greer Laboratories, Lenoir, NC) to wheat (8 mm X 20 mm) to standard reagent. Cow's Milk (7 mm X 20 mm), Egg (4 mm X 15 mm), Peanut (15 mm X 27 mm), Barley (5 mm X 15 mm), Rye (6 mm X 17 mm), Salmon (5 mm X 16 mm), Tuna (5 mm X 15 mm) Hazelnut (5 mm X 15 mm), Pecan (4 mm X 18 mm), Clam (5 mm X 21 mm), Crab Mix (5 mm X 25 mm), Shrimp (9 mm X 26 mm). During the visit, it was clear that additional foods needed to be identified so that her diet could be expanded, given her history of failure to thrive. As she had had no reaction history to wheat as an isolated food, the SPT size was similar to all the other food allergens tested, and the most recent exposure to wheat was distant (3.5 years prior), the decision was made to perform an OFC to wheat. Her family was aware of the risk involved with OFC, but perceived the benefit of reintroducing wheat into the diet to outweigh the risk of OFC.

A standard dosing protocol for IgE mediated allergy was followed for her wheat OFC (see Methods). She was administered a starting dose of 0.085 grams of wheat protein (1 g of wheat powder). She was asymptomatic. Twenty minutes after the second dose of 0.171 grams of wheat protein (2 g of wheat powder) was given, she developed emesis, rhinorrhea, cough, and wheeze. She was administered epinephrine (0.01 mg/kg of 1:1000 aqueous solution) i.m. She also developed diffuse hives, for which she was given cetirizine by mouth. Approximately 1 hour after this second dose of wheat protein was administered; she became pre-syncopal and had hypotension (lowest reading noted was 75/35 with a heart rate of 145). She was given supplemental oxygen via a face-mask. Additionally, a total of 1200 milliliters of normal saline was pushed via two peripheral intravenous catheters. Three more epinephrine injections i.m. were administered during the resuscitation, as she continued with hypotension despite the epinephrine and intravenous fluid administration. She was transferred to the Pediatric Intensive Care Unit for further treatment. While there, she had an additional episode of emesis. She was given an epinephrine infusion (0.02 mcg/kg/min) in the PICU for 4 hours. Adjunct medications given included ranitidine and methylprednisolone. The cumulative dose of wheat protein administered during this challenge was 256.25 mg.

This clinical case we presented shows, that wheat can cause severe and near-fatal anaphylaxis after ingestion of a low dose (˜260 mg). In this patient with established multiple food allergies, the previous reaction to wheat was distant in time, and it was not clear if it was due specifically to wheat or other food allergies (egg). The described OFC was done to confirm that a true IgE-mediated allergy existed. The food challenge was deemed necessary in face of slow growth of the patient due to a restricted diet for multiple food allergies. The patient had a skin test comparable to other foods to which she had known allergic reactions, which were distant in time. Wheat was chosen over egg and milk which are considered more dangerous than wheat challenge. After the severe reaction experienced during this OFC we reviewed our OFC cases in the last six years to see if wheat was associated with a higher risk of anaphylaxis, especially at low doses. No study before indeed has ever specifically analyzed the risk of wheat challenges. We divided the patients in 5 categories that represent progressively more severe reactions, with those requiring epinephrine for low dose antigen representing the more severe type. We hypothesized that factors that would contribute significantly to more severe reactions would display highest correlation for the more severe reaction such as those requiring the use of epinephrine. Using an univariate regression analysis we first identified in children clinical characteristics that were associated with the 5 different type reactions studied in children undergoing OFC to wheat vs. those children undergoing OFC to milk, egg, peanut, soy. In all studied groups, we found that children undergoing food challenges to wheat were younger and had smaller SPT. This is not surprising, as younger children tend to have a smaller size of skin testing. However, children undergoing a food challenge to wheat and those having any reaction had a higher sIgE to wheat. This is also to be expected as the cut-off levels for specific serum IgE for wheat are not well established, with most studies showing that children even with high levels of IgE may be able to pass a food challenge to wheat; moreover, given the importance of wheat in Western diets, parents and doctors are willing to accept higher risk of a failed OFC (4, 18, 52). However, children who experienced anaphylaxis to wheat did not have higher sIgE compared to children with a similar reaction to other foods (Table 1 and Additional file 1 Table S2E). Given the fact that sIgE was obtained in a small group of patients undergoing OFCs to wheat, we cannot exclude that this could be due to lack of statistical power. Indeed of 28 children who had multisystemic reactions for wheat, only 3 had measured levels of IgE.

Among children who reacted to wheat, we evaluated if any of the characteristics could predict a more severe reaction. We found that those who reacted with any type of reaction to wheat were more likely to have asthma (odds ratio [OR] = 3.2) and to have had a distant prior reaction to wheat (OR = 3.1). None of the studied characteristics were able to predict more severe reactions (Table 2). These findings could be due to the limited number of children (28 children, Table 1) having wheat-induced severe reactions.

We then analyzed with a univariate logistic regression which clinical characteristics, including undergoing a challenge to a specific food, were associated with higher risk of increasingly severe reactions (Table 3). As previously reported [6,7,52], the risk factors for OFCs resulting in reactions that needed epinephrine use were undergoing a peanut challenge, older age, being a male, having larger wheal and flare at SPT, higher sIgE levels, a history of prior reaction or prior reaction not limited to the skin. As with others before, we found that the strongest risk was actually represented by a having a prior reaction not involving the skin [7]. All the other ORs were close to 1, so they may be less clinically relevant. However the strength of their significance association was high and confirmed previously published data [3,5-7,15]. Moreover for the first time we showed that among children undergoing OFC for the main food allergens, wheat was more strongly associated than peanuts with the risk of having reactions requiring the use of epinephrine. This association was even stronger for those reactions that happened after a low dose ingestion, where OFC to wheat had a similar risk than having a prior reaction not involving the skin (Table 3).

A multivariate logistic regression analyzing the independent weight of the six factors associated more severe reactions showed that peanut challenges put patients at risk of anaphylaxis for low dose antigen (regardless of the use of epinephrine) and wheat challenges put patients more at risk for reactions requiring epinephrine use especially after low dose antigen (OR = 8.02) (Table 4). In our multivariate analysis we did not include sIgE, as they were not obtained for a substantial number of patients limiting their statistical power.

**Table 4:** Multivariate logistic regression to predict outcome of OFC with epinephrine use for low dose of allergen

	**Multisystemic**		**Multisystemic <1 g**		**Epinephrine**		**Epinephrine <1 g**	
	**OR**	**p**	**OR**	**p**	**OR**	**p**	**OR**	**p**
Wheat	1.3	**0.3**	1.5	**0.4**	2.4	**0.003**	8.02	**<0.0001**
Peanut	0.9	**0.9**	2.5	**0.02**	1.4	**0.1**	2.2	**0.03**
Soy	0.6	**0.1**	0.3	**0.1**	1.09	**0.8**	0.8	**0.8**
Age	1.01	**0.7**	0.9	**0.3**	1.08	**0.2**	1.01	**0.6**
Wheal mm	1.2	**<0.0001**	1.02	**0.6**	1.15	**<0.0001**	1.02	**0.07**
Prior Reaction not skin	1.8	**<0 *. *0001**	1.9	**0.04**	1.8	**<0.001**	1.4	**0.2**

Hence for the first time this paper identifies wheat challenge as an independent risk factor for severe reaction after OFC. These results could be explained by the relatively younger age of our patient population undergoing OFC to wheat, the lack of well defined risk factor such as cutoff levels to predict OFC failure, and the perceived safety in performing the food challenge to wheat compared to other foods. However, the intrinsic characteristic of the wheat allergen may predispose patients who have a true allergy to wheat to react with more severe reactions.

In our population, patients undergoing OFC to wheat were younger for all type of reactions. This could explain the more severe reactions, as younger children are less able to notify about early symptoms. However in the multivariate analysis, wheat OFC was associated with more severe reactions independent from age. Moreover, children undergoing wheat challenge may have had more severe allergy than those undergoing other OFC, as they had a higher sIgE to wheat than the children in the other groups. This can be explained by the fact that the sIgE or skin testing size cut off for wheat has poor specificity and sensitivity in predicting OFC outcomes [4], with most studies showing that children even with high levels of IgE may be able to pass a food challenge to wheat [4,18,53]. For example, Sampson et al. determined that 100 KU/L for wheat had a positive predictive value of only 75% [24]. Perry et al. reported that 56% of children with serum IgE >20 kU/L would pass the OFC to wheat. Similarly, Keet at al reported that children tend to outgrow wheat allergies, with a rate of resolution of 65% by 12 years of age. Although they reported that higher wheat IgE levels were associated with poorer outcomes, children outgrew wheat allergy with even the highest levels of wheat IgE. Indeed they reported that twenty-one percent of those whose peak wheat IgE level was more than 100 kU/L had resolved wheat allergy [18]. Also for skin testing, no reliable skin test cut off has ever been established [54,55]. The fact that specific IgE for wheat have poor positive predictive value for wheat allergy is due most likely to the cross reactivity between grass pollens and wheat [54-56]. Based on the above studies, most authors agree that oral re-challenges remain mandatory in case of allergy to wheat [53]. Hence, OFCs to wheat are often decided based on clinical history and necessity as well as the benefit/risk ratio to reintroduce a food. As wheat is a major staple of the Western diet, higher risk of failing OFCs is generally accepted by parents and physicians. Finally, physicians may have less experience with wheat challenges, as wheat allergy is less prevalent than egg, peanut milk and soy allergy and physicians may rely on the notion that wheat is a relatively safe OFC based on the data of the literature [15]. However wheat may pose a risk of severe reactions in patients truly sensitized and they may react to relatively low dose of antigen [54,55]. Furthermore, an increasingly high number of studies show a significant correlation of wheat ingestion and exercise induced anaphylaxis [9,10].

In conclusion, this study, for the first time, shows that wheat can be a risk factor for severe allergic reaction during OFC. It appears from our study that caution should be used in children undergoing OFCs to wheat who have had a previous history of a reaction that did not involve only the skin. Given the fact that children can react at a low dose, a starting dose of less than 100 mg should be advocated for wheat challenges. Recently, there have been two reports that indicate that omega-5 gliadin predicts for severe reactions in OFCs to wheat [47,48]. Thus, this marker could be used to select for those patients in whom OFCs should be done with extreme caution.

We acknowledge that the current study has the major limitation of being a retrospective study and the clinical data available for analysis (such as serum IgE levels) were not homogenously available for all patients as they were collected mainly based on clinical work up as directed by current guidelines for OFC and not based on research needs.

## Competing interests

The authors declare that they have no competing interests.

## Authors’ contributions

AC conducted data analysis, wrote the paper, KK conducted data extraction and data entry, RS did patient recruitment and wrote part of the paper, JF did patient recruitment, wrote part of the paper, JPG did data extraction and data entry, DN did data extraction and data entry, JS conducted data analysis, supervised project, created database. All authors read and approved the final manuscript.

## Supplementary Material

Additional file 1 Supplemental material. Click here for file
