# Supplementary material for: Oral food challenge to wheat: a near-fatal anaphylaxis and review of 93 food challenges in children
Source: World Allergy Organ J. 2013 Aug 21;6(1):14. doi: 10.1186/1939-4551-6-14 (PMC3765891; doi:10.1186/1939-4551-6-14)
Supplement: Additional file 1 — Supplemental material. [file 1939-4551-6-14-S1.docx]

**Supplemental material**

**Study population and oral challenges**

Informed consents were obtained from the participants and the study was approved by the Institutional Review Board of The Children Hospital of Philadelphia (CHOP), Philadelphia, PA. Children were initially seen in clinics at either the main hospital or its associated satellites offices. As previously reported, initial or repeated open food challenges were performed when oral tolerance was suspected (1E). Challenges were administered in escalating doses of the food allergen as tolerated every 20 minutes for a total age-appropriate cumulative dose by using an appropriate food or powdered protein (Barry Farm Enterprises, Wapakoneta, OH) camouflaged with juice, or other moist food, such as applesauce or pudding (1E, 2E). Hives secondary to direct contact with the food, such as those on the face or hands, were not considered positive reactions. Ambiguous reactions (25 total) were not included in this review. In patients with underlying atopic dermatitis, the skin was under good control at the time of challenge; patients’ skin was cleared with aggressive topical therapy and antibiotics if necessary prior to the challenge. Challenges were stopped if clear symptoms of an allergic reaction developed (3E). Emergency medications, including diphenhydramine, epinephrine, albuterol, and prednisone were administered at the provider’s discretion. Medication was prescribed for failed challenges on the basis of the type and severity of reaction. Short-acting antihistamine doses were diphenhydramine 1.5 to 2 mg/kg (maximum dose, 50 mg) orally. The same food challenge dose was repeated orally if the patient vomited within 30 minutes of receiving the dose. Epinephrine 0.01 mg/kg (maximum, 0.3 mg) per dose was administered intramuscularly every 15 to 30 minutes as needed to reverse symptoms. Albuterol 2.5 to 5 mg was also administered by nebulization for persistent chest symptoms. Prednisone 1 to 2 mg/kg (maximum dose, 60 mg) was given orally for refractory lower respiratory or gastrointestinal symptoms. The steroid dose was repeated if vomiting occurred within 30 minutes of the dose.

A negative or passed challenge was declared if patients ingested 100% of the intended dose without significant untoward effects. All patients were observed for a minimum of 2 hours or until signs of clinical reactivity subsided for those patients who failed the challenge. Patients were informed about late-phase reactions before discharge and instructed to contact the supervising physician immediately if symptoms recurred (3E). Briefly, OFC were performed by starting with a dose of 0.1 mL, followed by 0.5, 1, 2.5, 5, 10, 30, and 60 mL, 120 ml, and 240 ml for liquid foods (milk). For solid foods (peanut, egg powder, milk powder, wheat and soy), the challenge doses administered were 125 mg, 250 mg, 500 mg, 1 gm, 2 gm, 4 gm, 8 gm, and ad lib (minimum of 8 gm). In selected cases a lower starting dose (20-60 mg) was chosen for very high risk kids. Each dose was administered with an interval of 15 to 20 minutes until ad lib doses were reached or the patient experienced a reaction within 2 hours of the last dose. Challenges were stopped for gastrointestinal reactions, respiratory symptoms, non-contact cutaneous reactions or multi-system reactions (3E)

**Skin Testing**

Reactions of wheal and flare were recorded after 20 minutes by measuring the maximal longitudinal diameter of the wheal and the diameter orthogonal to it. Mean wheal diameters were calculated as (a + b)/2. A wheal of 3 mm greater than the negative control, accompanied by a flare, was considered positive. The positive control was 10 mg/mL of histamine dihydrochloride. Food specific IgE levels and SPTs were done within 6 to 12 month of OFCs.

**Statistical methods**

The children were categorized (positive or negative) on the basis positive OFC (any reaction), OFC that resulted in anaphylaxis (Anaphylaxis), OFC that resulted in anaphylaxis after low dose ingestion (Anaphylaxis <1g), OFC that resulted in anaphylaxis requiring epinephrine i.m. administration (Anaphylaxis with epi), and OFC that resulted in anaphylaxis requiring epinephrine i.m. administration after low dose ingestion(Anaphylaxis with epi<1g)

Collection of patient data was in compliance with the Institutional Review Board of The Children’s Hospital of Philadelphia.

|  | **TABLE 1E— Demographic of the study population** | | | | |
| --- | --- | --- | --- | --- | --- |
| **Characteristic** | Wheat (93) | Milk(290) | Egg(409) | Peanut(282) | Soy (114) |
| Age (mean ± SD) | 3.6 ± 2.4 | 4.4± 2.5 | 4.7± 2.5 | 6.1 ± 2.6 | 3.7 ± 2.7 |
| Sex (female/male) | 24 / 69 | 84/206 | 122/286 | 88/194 | 36 /78 |
| Wheal mm (mean ± SD) | 5.1 ± 2.6 | 6.5± 3.9 | 5.7± 3.3 | 6.3± 4.2 | 3.8 ± 2.4 |
| Flare (mean ± SD) | 12.1 ± 15.8 | 17.1± 8.8 | 16.6± 7.9 | 17.3± 10.0 | 10.1 ± 6.9 |
| Serum IgE (KUI/ml) | 19.9 ± 22.8 | 7.1± 14.7 | 4.8± 12.3 | 6.9± 18.8 | 14.6 ± 24.1 |
| Prior Ingestion | 57 | 262 | 296 | 174 | 68 |
| Prior Reaction | 50 | 248 | 247 | 163 | 56 |
| Prior Reaction not skin | 42 | 153 | 203 | 125 | 46 |
| Asthma History | 36 | 142 | 232 | 165 | 42 |
| Eczema History | 47 | 146 | 224 | 106 | 47 |
| Time of Prior Exposure ≥1 year | 34 | 200 | 208 | 146 | 38 |
| OFC starting dose <100mg | 4 | 5 | 2 | 1 | 0 |
| OFC positive | 39 | 144 | 189 | 130 | 26 |
| OFC positive for allergen <1g | 22 | 36 | 82 | 69 | 7 |
| Multisystemic reaction | 28 | 101 | 119 | 95 | 15 |
| Multisystemic reaction<1g | 17 | 23 | 56 | 56 | 3 |
| Epinephrine | 17 | 36 | 42 | 49 | 8 |
| Epinephrine<1g | 13 | 7 | 17 | 28 | 1 |

**Table 2E: Wheat sIgE and SPT to Wheat in relation to OFC outcome**

|  | **Food challenge to wheat negative (54)** | **Food challenge to wheat positive (39)** | **Multisystemic reaction(28)** | **Multisystemic reaction <1g (17)** |
| --- | --- | --- | --- | --- |
| **Wheat sIgE<0.35** | **0** | **0** | **0** | **0** |
| **Wheat sIgE<0.35-0.70** | **1** | **0 (0%)** | **0** | **0** |
| **Wheat sIgE 0.71-3.50** | **5** | **1 (16%)** | **0** | **0** |
| **Wheat sIgE 3.51-17.5** | **9** | **4 (30%)** | **1** | **1** |
| **Wheat sIgE 17.6-50** | **10** | **3 (23%)** | **1** | **1** |
| **Wheat sIgE 51-100** | **4** | **2 (33%)** | **1** | **0** |
| **Skin Prick test wheal 0-2 mm** | **11** | **6 (35%)** | **0** | **0** |
| **Skin Prick test wheal 3-5 mm** | **29** | **19 (39%)** | **12** | **11** |
| **Skin Prick test wheal 6-8 mm** | **7** | **8 (53%)** | **4** | **2** |
| **Skin Prick test wheal ≥9 mm** | **4** | **4 (50%)** | **1** | **0** |
| **Age (Mean±SD)** | **3.7±2.8** | **3.3±1.7** | **3.5±1.7** | **2.8±1.2** |
| **Prior reaction not skin** | **20** | **22** | **14** | **8** |

**Table 3E: Characteristics of children undergoing open oral food challenge (OFC) to wheat vs those undergoing OFC to other foods**

| **Type of Children** | **Mutlisystemic reaction<1g** | | | **Epinephrine<1g** | | |
| --- | --- | --- | --- | --- | --- | --- |
|  | Wheat (17) | Other  (138) | p | Wheat (13) | Other  (53) | p |
| Age  (mean ± SE) | 2.8 ± 0.29 | 5.1± 0.20 | **<1X10^-3^** | 2.9 ± 0.4 | 5.5 ± 0.3 | **<1X10^-3^** |
| Sex (female)  (5) | 2  (11%) | 43 (31%) | **0.09** | 1  (8%) | 17 (32%) | **0.2** |
| Wheal mm (mean ± SE) | 4.5 ± 0.51 | 8.5 ± 0.32 | **<1X10^-4^** | 4.6 ± 0.4 | 9.4 ± 0.6 | **<1X10^-3^** |
| Flare mm  (mean ± SE) | 11.9±  1.15 | 21.8 ± 0.7 | **<1X10^-4^** | 12.5±1.7 | 24.4 ± 1.2 | **<1X10^-4^** |
| sIgE (KUI/L)  (mean ± SE) | $23± 18 | $$13.1 ± 2.2 | **ns** | 41±n/a | 17.8 ± 5.1 | **ns** |
| Prior  Ingestion | 10  (59%) | 96 (70%) | **ns** | 8  (62%) | 34 (63%) | **ns** |
| Prior  Reaction | 10  (59%) | 95 (69%) | **ns** | 8  (62%) | 34 (64%) | **ns** |
| Prior  Reaction not skin | 8  (47%) | 76 (55%) | **ns** | 6  (46%) | 31 (58%) | **ns** |
| Asthma History | 10  (59%) | 85 (61%) | **ns** | 8  (62%) | 30 (56%) | **ns** |
| Eczema History | 10  (59%) | 71  (52%) | **ns** | 8 (62%) | 28  (52%) | **ns** |
| Time of Prior Exposure ≥1 year | 8  (47%) | 84  (61%) | **ns** | 7  (54%) | 34  (64%) | **ns** |

**References**

E1. Nowak-Wegrzyn, A., Assa'ad, A.H., Bahna, S.L., Bock, S.A., Sicherer, S.H., and Teuber, S.S. 2009. Work Group report: oral food challenge testing. *The Journal of allergy and clinical immunology* 123:S365-383.

E2. Perry, T.T., Matsui, E.C., Conover-Walker, M.K., and Wood, R.A. 2004. Risk of oral food challenges. *J Allergy Clin Immunol* 114:1164-1168.

E3. Spergel, J.M., Beausoleil, J.L., Fiedler, J.M., Ginsberg, J., Wagner, K., and Pawlowski, N.A. 2004. Correlation of initial food reactions to observed reactions on challenges. *Ann Allergy Asthma Immunol* 92:217-224.
